# Diagnosis of an extremely rare pleomorphic adenoma of the breast with core needle biopsy: A case report

**DOI:** 10.1016/j.amsu.2018.10.037

**Published:** 2018-11-05

**Authors:** Keiichi Takahashi

**Affiliations:** Takahashi Breast and Gastroenterology Clinic, Yamazaki Seiren Bldg. 2F, 6-2-22, Uehonmachi, Tennoji-Ku, 543-0001, Osaka, Japan

**Keywords:** Pleomorphic adenoma, Subareolar area, Bone or cartilage formation, PA, Pleomorphic adenoma, PAB, Pleomorphic adenoma of the breast, CNB, Core needle biopsy, MMG, Mammography, US, Ultrasonography

## Abstract

**Introduction:**

Pleomorphic adenoma (PA) rarely originates in the mammary gland. This tumor is a benign mixed tumor and is commonly found in the salivary glands, but rarely shows findings similar to those of breast cancer. The tumor is misdiagnosed in 30–50% of the patients, including overdiagnosis of malignancy preoperatively, leading to unnecessary surgery for breast cancer.

**Case presentation:**

The present patient was a 45-year-old woman who exhibited no subjective symptoms. She visited the Takahashi Breast and Gastroenterology Clinic for breast cancer screening. A mass measuring 1.5 cm was palpated in the E region of the left breast. Mammography (MMG) showed a tumor shadow in the left S region. There was a high-density area inside the tumor, suggesting bone or cartilage formation, and a diagnosis of category 3 on MMG was made. Ultrasonography (US) revealed a poorly demarcated tumor measuring 14.3 × 14.8 × 10.7 mm with relatively smooth margins and inhomogeneous high-echo content. Core needle biopsy (CNB) was performed, which led to the diagnosis of PA. The tumor showed proliferation of small cells forming glandular duct-like structures or anastomosed funicular structures. Moreover, the cells in the basilar portion were spindle shaped and transitioned to the stroma. Cartilaginous metaplasia, calcification, and ossification were observed in some areas. Intraductal papilloma was also observed in some areas.

**Conclusion:**

PA may be diagnosed based on the histological findings of CNB. Thus, unnecessary surgery for breast cancer may be avoided.

## Highlights

• Pleomorphic adenoma of the breast is extremely rare and a benign tumor that frequently occurs around the subareolar area.
• Pleomorphic adenoma of the breast is often misdiagnosed as breast cancer and surgically treated as a malignant tumor.
• Insufficient excision results in not only local recurrence, but also malignant conversion.
• Pleomorphic adenoma of the breast may be dignosed on the histological findings of core needle biopsy.
• Thus, unnecessary surgery for breast cancer may be avoided.

## 1 Introduction

This work has been reported in line with the surgical case report (SCARE) criteria [1].

Pleomorphic adenoma (PA) is a benign mixed tumor often occurring in the salivary gland, and rarely originating in the breast [[2], [3], [4], [5], [6]]. This tumor consists of epithelial and myoepithelial components. Bone or cartilage formation is a characteristic feature of PA. Differential diagnosis include fibroadenoma, phyllodes tumor, metaplastic cancer, and mucinous carcinoma. Most important, PA of the breast (PAB) could be misdiagnosed as a primary sarcoma or metaplastic carcinoma of the breast owing to the abundance of metaplastic stroma [3,7,8]. Therefore, this tumor can rarely present findings similar to those of breast cancer, 30–50% of patients are initially misdiagnosed as having malignant disease preoperatively [[9], [10], [11]] and undergo unnecessary surgery such as total, radical, or modified mastectomy for breast cancer [[2], [3], [4], [5], [6]]. Herein, a case of PA identified with core needle biopsy (CNB) is reported.

## 2 Case presentation

**Fig. 1:**
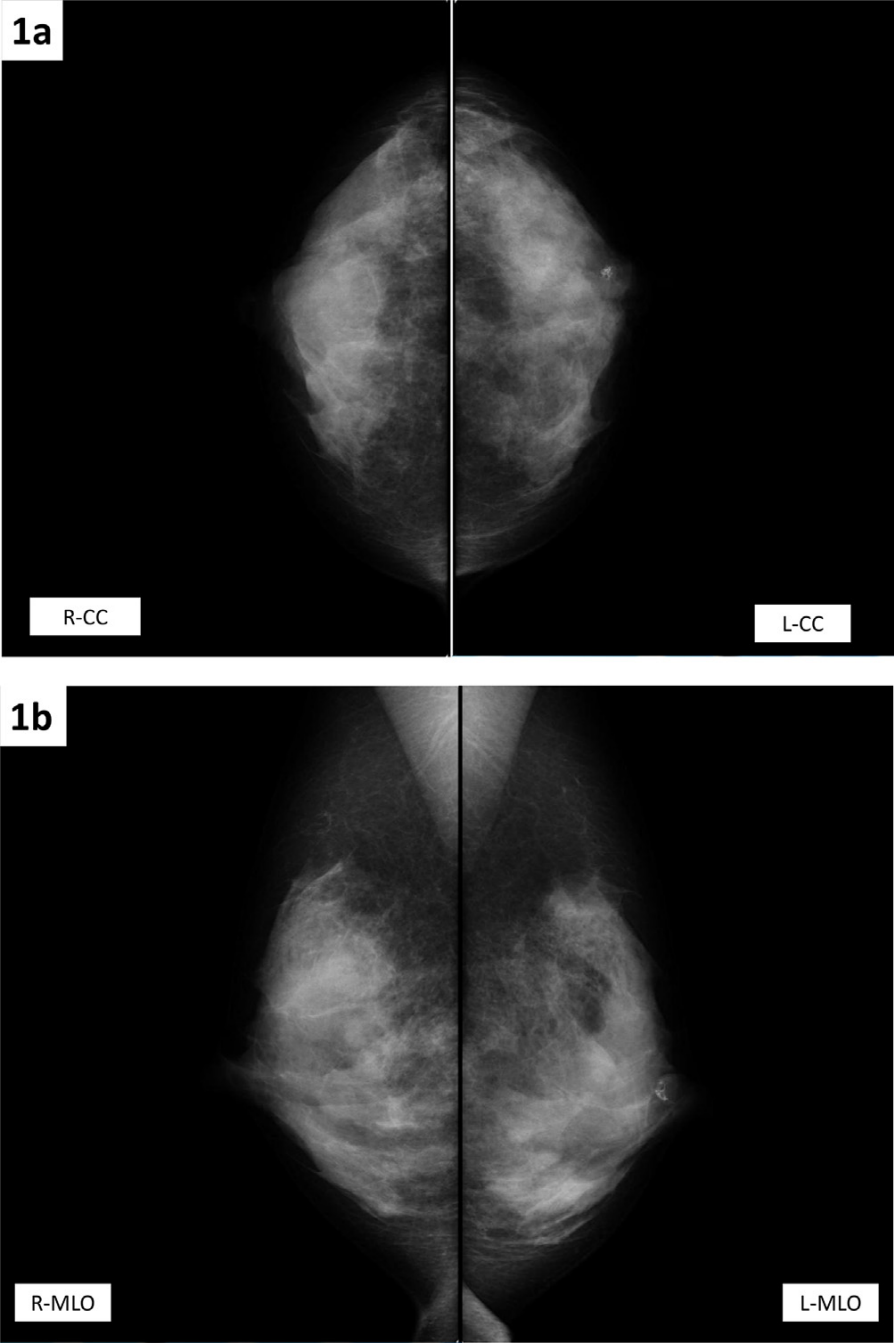
**Mammography; 1(a,b)**. Mammography showed a tumor shadow in the left S region. There was a high-density area inside the tumor, suggesting bone or cartilage formation, and a diagnosis of category 3 for mammography was made.

**Fig. 2:**
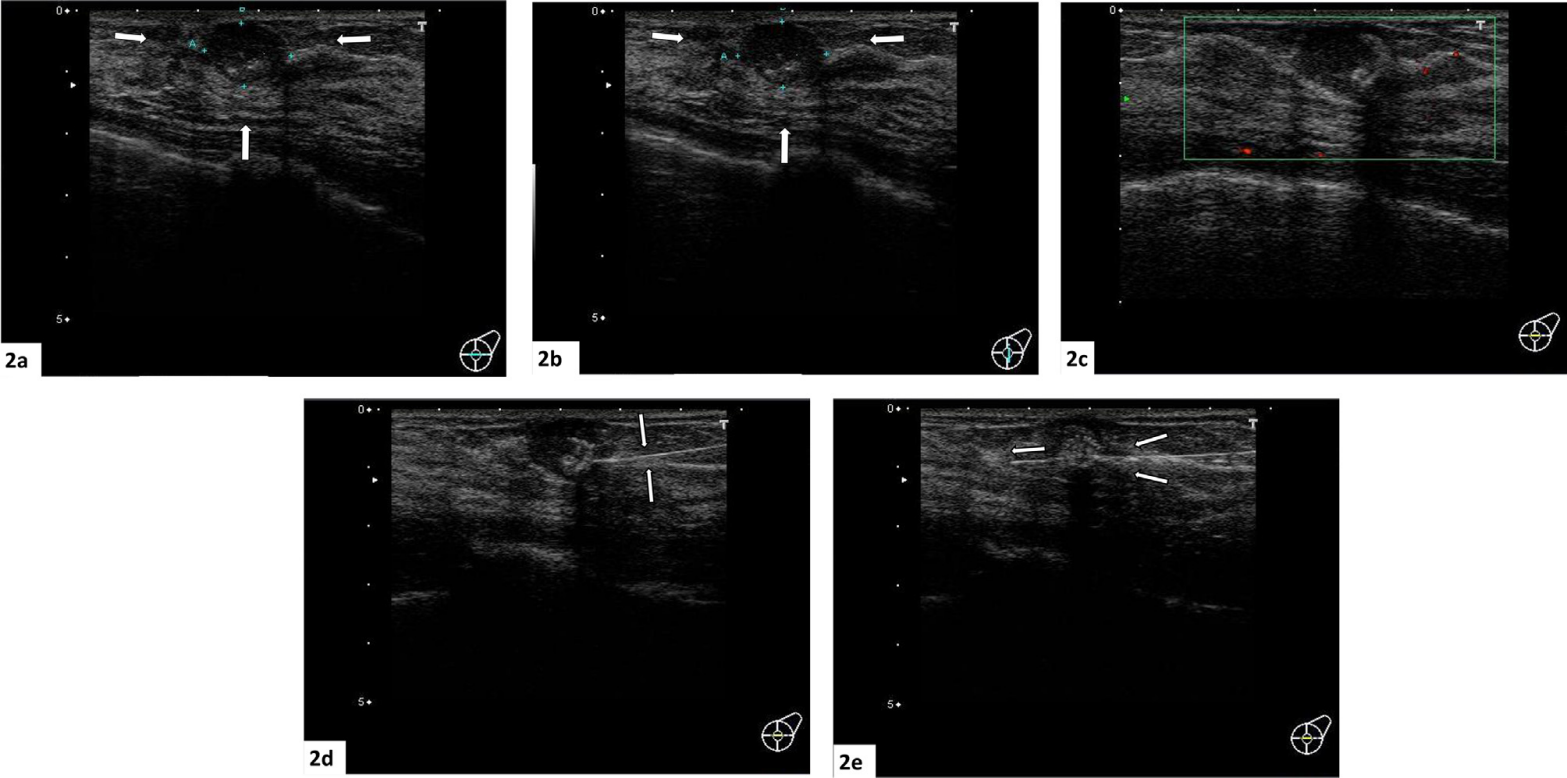
**Breast ultrasonography; 2(a,b,c)**. Ultrasonography revealed a poorly demarcated tumor measuring 14.3 × 14.8 × 10.7 mm with relatively smooth margins and inhomogeneous high-echo content (Fig. 2a and b). Color Doppler US showed a hypovascular pattern (Fig. 2c). (For interpretation of the references to colour in this figure legend, the reader is referred to the Web version of this article.)

The present patient was a 45-year-old woman who exhibited no subjective symptoms. She visited the Takahashi Breast and Gastroenterology Clinic for breast cancer screening. A mass measuring 1.5 cm was palpated in the E region of the left breast. MMG showed a tumor shadow in the left S region. There was a high-density area inside the tumor, suggesting bone or cartilage formation, and a diagnosis of category 3 on MMG was made (Fig. 1a and b). US revealed a poorly demarcated tumor measuring 14.3 × 14.8 × 10.7 mm with relatively smooth margins and inhomogeneous high-echo content (Fig. 2a and b). Color Doppler US showed a hypovascular pattern (Fig. 2c). CNB was performed. The linear shadow passing from the right side transversely and slightly obliquely downwards, anterior to the tumor was the needle used in the biopsy (Fig. 2d). The needle passed through the solid tumor steadily and accurately (Fig. 2e).

**Fig. 3:**
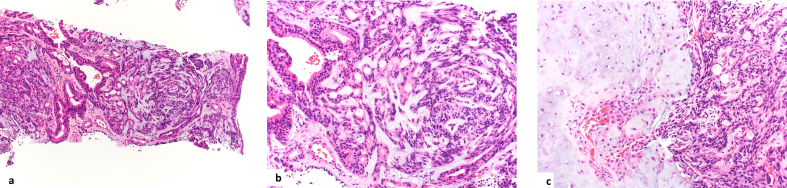
**Pathological analysis; 3(a,b,c). (3a:**【**H-Estain,x100】, 3b:【H-Estain,x200】, 3c:【H-Estain,x200**】). The pathological findings of the CNB specimen indicated pleomorphic adenoma. The tumor showed proliferation of small cells forming glandular duct-like structures or anastomosed funicular structures, and the cells in the basilar portion were spindle-shaped and transitioned to the stroma. Cartilaginous metaplasia, calcification, and ossification were observed in some areas. Intraductal papilloma was also observed in some areas. Diagnosis of pleomorphic adenoma was made based on the histological picture from CNB.

The pathological findings of the CNB specimen indicated PA. The tumor showed proliferation of small cells forming glandular duct-like structures or anastomosed funicular structures, and the cells in the basilar portion were spindle-shaped and transitioned to the stroma. Cartilaginous metaplasia, calcification, and ossification were observed in some areas. Intraductal papilloma was also observed in some areas. The diagnosis of PA was made based on the histological picture of CNB (Fig. 3a, b, 3c).

## 3 Discussion

PA accounts for 55–70% of all salivary gland tumors. It also occurs in the skin, larynx, and palate and maxillary sinus and nasal seputum and tracheobronchus and lung and lacrimal gland. It rarely occurs in the mammary gland [[2], [3], [4], [5], [6]]. Most patients with this tumor are women ranging in age from 23 to 78 years, and the frequent site of involvement is around the subareolar area [[2], [3], [4], [5], [6]]. Typically, this tumor is clearly demarcated from the surrounding tissue, often has fibrous capsules, and is characterized by the coexistence of epithelial and mesenchymal components, showing various histological features including the glandular duct, myoepithelial cells, myxomatous stroma, and cartilage component. The mammary gland is a compound tubuloalveolar gland comprising myoepithelial cells and resembles the salivary gland. Cells and matrix of bone and cartilage formation distinguish it from fibroadenoma accompanied by myxomatous stroma or phyllodes tumor [[2], [3], [4], [5], [6]].

PA is likely to occur in the adjacent subareolar area. The reason is thought to be because myoepithelial cells are distributed abundantly around the mammary ducts of the subareolar area [12,13].

The histogenesis of mixed breast tumors is considered to resemble that of tumors of the salivary glands [14,15].

PAB may originate and develop from an intraductal papilloma [14,17,18].

The myoepithelial cells of papilloma are extraordinarily stimulated and therefore grow to form the characteristic stromal elements. It has also been suggested that multifocal PA may develop from multiple intraductal papilloma [14].

In imaging findings, PA is often depicted as a tumor of a uniform concentration with a clear boundary [6], sometimes irregular in shape, and has no calcifications [4] or microcalcifications on MMG. Moreover, there are cases exhibiting findings suggestive of suspected malignancy, such as a mass with unclear boundary, numerous coarse, or diffuse irregular central calcifications, and densely and partly calcified appearance [2,5,12,19]. US findings were reported to show a homogeneously smooth, or lobulated internal mass, and the posterior echo is often enhanced.

In cytological findings, epithelial-like cells similar to plasma cells and a myxoma component with orbicular-ovate or spindle shaped cells are observed. Moreover, although there is no atypia of epithelial cells, cells that show polymorphism are sometimes recognized in the myxomatous part, whereas nuclear mitosis has been reported to be hardly seen. However, it is difficult to diagnose PA which shows a variety of tissue imaging findings with cytology [15] or CNB alone [2]. Many reports have shown the difficulty of the differential diagnosis between benign and malignant disease before resection owing to the abundance of metaplastic stroma [3,7,8].

In addition, it is difficult to diagnose PAB with intraoperative frozen section of the resected tissue [2,5,15]. PAB is often misdiagnosed as a malignancy such as metaplastic carcinoma on frozen section examination [5]. With respect to the operative procedure, many patients undergo overaggressive surgery, including total, radical, or modified mastectomy because of the suspicion or misdiagnosis of malignancy [3,5,15].

When surgical resection is performed, care should be taken not to break the tumor capsule. It is recommended to perform a local resection with adequate margins of more than about 3mm cuff of normal tissue [2,20]. Damage to the capsule during surgical resection may lead to seeding of tumor cells, and multi-focal growth is sometimes recognized [14,[16], [17], [18], [19],^21^,^22^]. Since PAB frequently occurs in the periareolar region [12,13], a sufficient margin cannot be taken when nipple sparing surgery is performed, which is a risk factor for local recurrence. The recurrence site is mostly in the adjacent subareolar area, the median recurrence time is 4 years after surgery, and an elapsed observation time of at least 5 years is recommended [2,23].

Insufficient excision results in not only local recurrence, but also malignant conversion [24]. Therefore, complete excision of the tumor must be achieved.

## 4 Conclusion

PAB is a benign tumor that frequently occurs around the subareolar area. It is often misdiagnosed as breast cancer and surgically treated as a malignant tumor. Insufficient excision results in local recurrence or malignant conversion. For its treatment, it is important to excise the tumor while maintaining the tumor capsule intact and maintaining an adequate distance from the cut end. If there is doubt about the case, the tumor should be removed from a distance of more than 5mm, and the diagnosis should be made according to the results of a permanent specimen of the fully excised tumor.

When a tumor with several histological features is detected around the subareolar area, it is important to establish diagnosis and perform treatment with caution while considering a possible differential diagnosis of PAB, despite the extreme rarity of this tumor.

## Ethical approval

Not applicable.

## Sources of funding

None.

## Author contribution

Keiichi Takahashi performed the procedure, wrote the manuscript and is responsible for the information.

## Conflicts of interest

None.

## Research registration number

Not applicable.

## Guarantor

Keiichi Takahashi is the guarantor of this paper.

## Patient consent

The patient provided her informed consent for the publication of her clinical details and any accompanying images about this case report.

## Provenance and peer review

Not commissioned, externally peer reviewed.

## Appendix A Supplementary data

Checklist Checklist
